# Design of novel DC-DC interleaved boost converter for BLDC application

**DOI:** 10.1016/j.heliyon.2024.e40041

**Published:** 2024-11-04

**Authors:** P.M. Preethiraj, J. Belwin Edward

**Affiliations:** Vellore Institute of Technology, Vellore, 632014, Tamilnadu, India

**Keywords:** DC-DC converter, Fuel cell, Interleaved boost-cuk converter, BLDC, Voltage regulations, Current ripple, Efficiency

## Abstract

The gradual increment of renewable energy sources (RES) integration in the power generation system is involved in achieving the maximum power demand in the world. In RES fuel cells are used for storage systems of energy. The increment and decrement of voltage level can be controlled by the different types of converters as per the application. Based on that researchers have introduced different types of converters like interleaved converters for voltage-boosting applications to operate the drive. But the converters are involved with higher parts count, switching losses, efficient speed regulation, and torque ripple. To bridge the research the challenges involved in design and operation of converter new interleaved converter structure has been proposed. The validation of proposed converter has been performed in environment of MATLAB/SIMULINK and the required characteristics of the converter and BLDC have been presented. To validate constructional and operational features of converter. The hardware has been developed for 1300 W and operational characteristics have been presented

## 1 Introduction

Almost all of the electricity produced is the product of conventional energy sources: petroleum, coal, or natural gas. These sources lead to the production of carbon dioxide (CO2) and are largely responsible for the process of global warming. Burning fossil fuel is a major driver for climate change since it produces greenhouse gases the emission of GHG into the atmosphere. Due to fossil fuels, approximately 80% of GHGs are produced Taghvaee et al. [1]. Because of this, it is becoming more and more important to create green energy sources as a way to provide clean energy with no pollution. Finding renewable energy is crucial. It uses renewable energy sources, like fuel cells, wind power and solar photovoltaics; Motor drives, uninterruptible power systems, electric vehicles, and microgrids are among the applications that are becoming more prevalent Li et al. [2]. Green hydrogen may reduce carbon footprints and promote sustainability in transportation and energy. Buberger et al. [3], Oliveira et al. [4]. Because FCs are electrochemical devices that use an electrolytic technique to transform chemical processes into electrical energy, only heat, and water are produced as byproducts, making them major participants in the global energy market.

**Figure 1:**
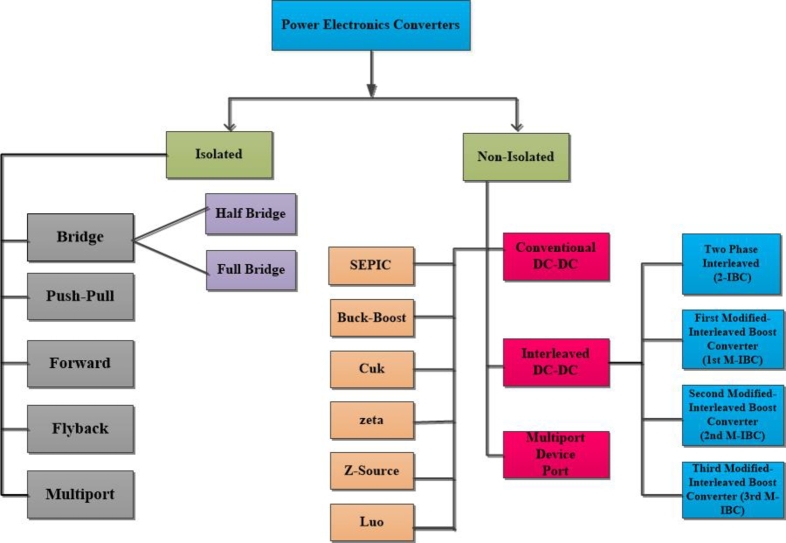
Boost converter topologies.

There are several categories of fuel cells, such as alkaline fuel cells, phosphoric fuel cells, molten carbonate fuel cells, solid oxide fuel cells, combined heat and power fuel cells, proton exchange membrane fuel cells, and regenerative fuel cells. Each reversible fuel cell has distinct properties determined by its use's specific nature Karami et al. [5]. PEMFC fuel cells are most common. The primary reasons for this are its exceptional efficiency, minimal emissions, minimal noise output, and reduced operating temperature. Nevertheless, Proton Exchange Membrane Fuel Cell (PEMFC) devices have a sluggish power response and an uncontrolled direct current (DC) voltage output. The inherent complexity of the mechanical design, together with the electrochemical features of its polarization curve, contribute to this phenomenon. The performance of the electrochemical process and the resulting voltage in a fuel cell may be influenced by several operating variables, including temperature, pressure, relative humidity, water, air, and hydrogen. At these operating points, implementing power converters would be necessary to connect the fuel cell to the load. Khan et al. [6]. By acting as a middleman between source of power and load, power electronic converters enable input voltage to be adjusted to suit the needs of the application. In power engineering and driving, power converters have been widely used for many years. Now serve as substitutes for more traditional power conversion and rheostat circuits, which split voltage Sivakumar et al. [7]. Conventional approaches often exhibit an output voltage and efficiency is very low. The concept of DC-DC converters was first introduced in the 1920s. Over the last six decades, these converters have been extensively used in many applications and have significantly contributed to the field of PE and drives. They have wide-ranging uses in many industrial sectors and computer electronic circuits and play crucial role in the advancement of renewable energy technologies. Converters play a crucial role in hybrid renewable energy systems by providing voltage stabilization during intermittent conditions. The power converter's operational stability and control methodology significantly influence the power distribution quality in renewable energy systems Kolli et al. [8], Mumtaz et al. [9]. DC-DC converters typically categorized into 2 main types: isolated and non-isolated. Non-isolated converters can be categorized based on whether they possess a shared ground or a floating ground. An isolated converter ensures that there is complete electrical separation between the input and load terminals Several research publications have proposed a unidirectional DC-DC converter with high gain and high efficiency for diverse applications, including renewable energy and electric cars. These converters are specifically designed to provide the higher voltages required for some electric vehicle charging applications. Fig. 1 demonstrates that both isolated and non-isolated converters may be categorized into two types: unidirectional or bidirectional converters Venugopal et al. [10]. The isolated converters include Multilevel Boost Converters (MLBC), Three-Level Boost Converters (TLBC), Interleaved Boost Converter (IBC), Positive Output Super-Lift (POSL), and Single-Ended Primary Inductor Converters (SEPIC). To maximize output voltage of fuel cell (FC), Voltage gain ratio and voltage stress are primary performance indicators of the converters that must be assessed Rosas-Caro et al., Rosas-Caro et al., ˙INċ et al., Alsafrani et al., Alsafrani et al., Youn et al., Zhang et al., Elsayad et al., Nahar et al., Harinee et al., Chen et al., Jang et al., Zhou et al., Varesi et al., Fang Lin et al., Berkovich et al., Mohammed et al., Gholizadeh et al., Mahdizadeh et al., Verma et al., Gu, W et al., Gules et al., Ozsoy et al., Kumar et al., Ahmed et al. [11], [12], [13], [14], [15], [16], [17], [18], [19], [20], [21], [22], [23], [24], [25], [26], [27], [28], [29], [30], [31], [32], [33], [34], [35]. Literature review on different converters based on Fuel cell shown in Table 1.

**Table 1:** Literature review.

Author/Ref	Converter	Merits	Demerits
Kumar, K., et al. [36]	Quadratic Boost	• High voltage gain transfer	• Decreases general proficiency of the system.
Farhani, Slah, et al. [37]	Interleaved Boost	• Minimized current pressure. • Reduced Current ripple	• Component stress owing to increased components.
Girirajan, Balasubramanian, et al. [38]	High Gain Converter	• Conversion ratio of converter is improved, which reduces current ripple and voltage stress.	• High switching loss occurs. • Slow response.
Kiran, Shaik Rafi, et al. [39]	Single Switch Boost	• Stress across power switches is low. • Achieves continuous power supply.	• Power gain is low and requires more switches.
Shilaja, C., et al. [40] 2022	Boost Converter	• Good response with high tracking speed. • Less conduction and increased voltage gain ratio.	• Switches receive an excessive amount of current. • Voltage is extremely sensitive to changes in duty cycle.

**Motivation & Research gap:**

There are many research gaps in the existing literature, such as solutions that are not as advanced as the latest ones and are not good enough for real-world testing in changing conditions with good voltage conversion and motor control for fuel cell-powered electric vehicles. Furthermore, the extent to which present approaches may be scaled and adapted, as well as their dynamic performance, have not been thoroughly investigated. This drives the development of advanced fuel cell technology due to its exceptional efficiency and minimal emissions, which are crucial for sustainable energy solutions. Improved performance and dependability of fuel cell electric vehicles (EVs) are essential for increasing driving distances and advancing sustainable energy use. Make sure it can be used in the real world and is reliable by running a lot of simulations and checking it out in the real world by combining effective voltage conversion and reliable motor control into a single system.

The objective of paper is to enhance regulation of a Brushless DC (BLDC) motor system via the use of a specifically engineered power converter and control approach. A comprehensive explanation of each contribution is provided below.

Interface for Interleaved Boost-Cuk Converter.

A hybrid power converter containing characteristics of both Boost and Cuk topologies is presented in the study, known as Interleaved Boost-Cuk converter. An essential objective of this converter is to elevate output voltage from fuel cells (FCs) to a more stable and higher level, necessary for operating the BLDC motor.

An interleaved design, which involves parallel converters running with phase shifts, serves to decrease current ripple, enhance efficiency, and evenly distribute the power load. Consequently, Comparatively to conventional converters, this design increases the system's dependability and efficiency. Accurate Speed Regulation for Brushless DC Motor using Primary Inverter Controller.

A Proportional-Integral (PI) controller is included in system to control the speed of Brushless DC (BLDC) motor. A proportional-integral (PI) controller regulates the input voltage of the motor by measuring the discrepancy between the intended and motor speeds. Correcting for disturbances and load fluctuations, this guarantees steady operation. Outcome is a seamless and effective regulation of motor speed, avoiding overshooting or instability, prevalent problems in motor control systems without feedback mechanisms. Computational simulations via MATLAB.

The system under consideration has undergone comprehensive testing inside a MATLAB simulation environment, a commonly used tool for power electronics and control system design. These simulations assess important performance indicators like voltage regulation, motor speed control, power efficiency, and general system stability. The findings indicate substantial improvements in efficiency and improved power tracking, therefore enabling the system to provide power to the motor more efficiently than conventional approaches, such as using basic DC-DC converters or less rigorous control techniques.

Organization of this paper is as follows: Section 2 explains fuel cell model, Section 3 presents a correlation between the converters, and a detailed explanation of the converter that is being suggested and the simulation findings in Section 4.

## 2 Model of fuel cell

Various kinds of fuel cells are available for the automobile industry, including Proton Exchange Membrane Fuel Cells (PEMFC), Phosphoric Acid Fuel Cells (PAFC), Alkaline Fuel Cells (AFC), and Solid Oxide Fuel Cells (SOFC), Molten Carbonate Fuel Cell (MCFC) Pathak et al. [41]

A better understanding of the various models of fuel cells information has been tabulated in Table 2 for the purpose of design, the authors have proposed the mathematical evaluation for fuel cells from equation

(1) $$V_{\text{fuel}}=N_{\text{cell}}\cdot V_{\text{SFC}}$$

(2) $$V_{\text{fuel}}=N_{\text{cell}}\cdot (E_{\text{Nernst}}-V_{\text{act}}-V_{\text{ohmic}}-V_{\text{conc}})$$

where, $V_{Fuel}$ Fuel cell output voltage, $N_{cell}$ No. of cells, $V_{SFC}$ Voltage of single fuel, cell $E_{Nernst}$ nernst voltage $V_{act}$ activation loss $V_{Ohmic}$ is Ohmic loss $V_{Conc}$ concentration loss

(3) $$E_{\text{Nernst}}=[1.229-8.5\times 10^{-4}\left(T-298.5+4.308\times 10^{-5}\cdot T\cdot (\ln(P_{{\text{H}}_{2}})+\ln(P_{{\text{O}}_{2}}))\right)]$$

where, $T_{abs}$ absolute temperature, $P_{{\text{H}}_{2}}$, $P_{{\text{O}}_{2}}$ is Partial pressure of hydrogen and oxygen

(4) $$V_{\text{act}}=k_{1}+k_{2}\cdot T_{\text{abs}}+k_{3}\cdot T_{\text{abs}}\cdot \ln(CO_{2})+k_{4}\cdot T_{\text{abs}}\cdot \ln(I_{\text{fuel}})$$

where $k_{1}$, $k_{2}$, $k_{3}$ & $k_{4}$ is semiempirical coefficient, $I_{fuel}$ fuel cell current

(5) $$V_{\text{conc}}=\frac{R_{T}}{nF}\ln\left[1-\frac{I_{\text{fuel}}}{I_{\text{lim}}A_{\text{c}}}\right]$$

where R is resistance, F is Faraday's constant $I_{lim}$ is limiting current

(6) $$R_{\text{ohms}}=\frac{r_{\text{rm}}\cdot t_{m}\cdot t}{A_{c}}$$

where, $r_{rm}$ is resistivity of membrane $t_{m}$ thickness of the membrane $A_{c}$ Active cell area.

**Table 2:** Differences of the fuel cell.

	PEFC	AFC	PAFC	MCFC	SOFC
Electrolyte	Hydrated Polymeric ion Exchange Membranes	Mobilized or immobilized Potassium Hydroxide in asbestos matrix	Immobilized Liquid Phosphoric Acid in SiC	Immobilized Liquid Molten Carbonate in LiAlO2	Perovskites
Electrodes	Carbon	Transition metals	Carbon	Nickel & Nickel Oxide	Perovskite & perovskite/ metal cermet
Catalyst	Platinum	Platinum	Platinum	Electrode material	Electrode material
Interconnect	Carbon or Metal	Metal	Graphite	Stainless steel or Nickel	Nickel, Ceramic, or steel
Operating Temperature	40-80 ^∘^C	65-220 ^∘^C	205 ^∘^C	650 ^∘^C	600-1000 ^∘^C
Charge carrier	H^+^	OH^−^	H^+^	CO3^−^	O^−^

Based on the equation, fuel cells in Niu et al. [42], Becherif et al. [43].

**Figure 2:**
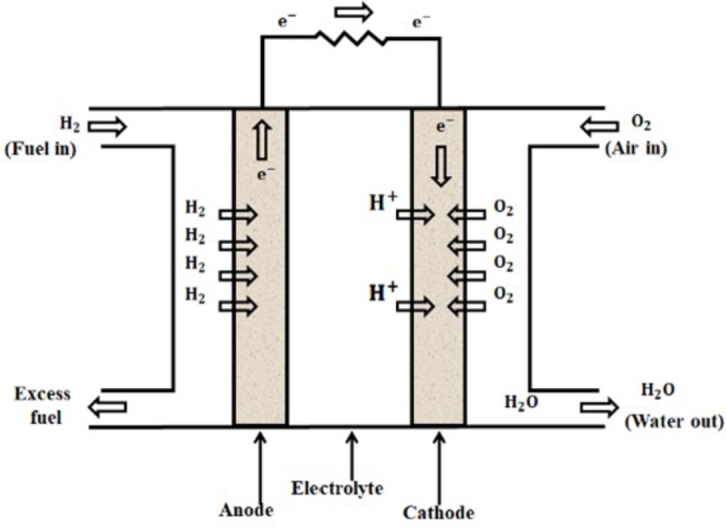
Construction of PEM Fuel Cell.

The Proton Exchange Membrane Fuel Cell (PEMFC) is gaining popularity due to its adaptability, high efficiency, and power density. Compared to energy converters that rely on traditional fossil fuels for electricity generation, PEMFC is more efficient. Fig. 2 illustrates the architecture of Proton Exchange Membrane Fuel Cell (PEMFC) Hydrogen ions function as transporters in PEMFC, transferring from the anode to the cathode. The anode compartment is supplied with hydrogen gas. As a result of chemical composition of H2, hydrogen ions (H+) and electrons (e-) get detached from each other. Subsequently, the positively charged hydrogen ions (H+) traverse the polymer membrane electrolyte and reach the cathode. Due to the prohibition of traveling through the proton exchange membrane, electrons are required to traverse an external circuit in order to transition from anode to cathode. The cathode chamber simultaneously receives air containing both positively charged H+ ions and negatively charged electrons. Water molecules form by the combination of oxygen in the air with e and H+ ions. The chemical reaction that occurs during operation is as follows:

(7) $$\text{Anode Reaction: }H_{2}\to 2H^{+}+2e^{-}$$

(8) $$\text{Cathode Reaction: }2H^{+}+\frac{1}{2}O_{2}+2e^{-}\to H_{2}O$$

(9) $$\text{Overall Reaction:}H_{2}+\frac{1}{2}O_{2}\to H_{2}O$$

**Figure 3:**
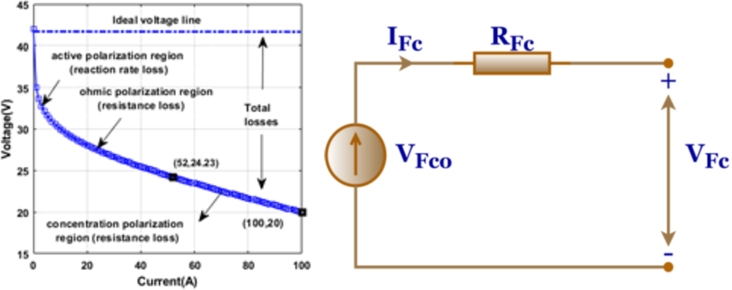
Fuel cell, (a) I-V Curve (b) equivalent circuit of FC system.

**Fuel Stack:** Due to the insufficient voltage generated by a single cell, often ranging between 0 and 1 V, necessary to connect numerous cells to create a fuel cell stack capable of efficiently powering semiconductor devices. A fuel cell (FC) can produce sufficient power for small appliances. However, for larger power requirements, it must be supplemented with other energy sources such as supercapacitors or rechargeable batteries. Minimizing the produced DC voltage caused by the internal resistance of fuel cell may be achieved by quickly changing the load current. When the system experiences sudden power dips, it is common to use extra storage in combination with a fuel cell Srinivasan et al., Alavi et al. [44], [45]. Convenient uses of fuel cells (FCs) offer several benefits over ordinary batteries due to their distinct properties and much better power density, which is 5 to 10 times more. Portable fuel cells can generate a range of power. The manufacturer Horizon has previously introduced instructional remote-control toys, kits, and equipment to the market, which includes an instructive package for a hydro vehicle. Electricity is generated via the use of portable power generators based on fuel cells when it is not feasible to connect to a power grid. The portable generator seems sufficient for personal outdoor usage, security, and catastrophe help. Fuel cell technology has significant importance in the stationary power generating industry. Fig. 3 illustrates the layout of the equivalent circuit for a PEMFC. The term “V${}_{F0C}$” represents the open-circuit voltage and includes internal resistance as well. A diode is used in series with the output to regulate the influx of negative current into the system Reddy et al. [46].

## 3 Interleaved boost converter (IBC)

Interleaved structure is an effective technique for increasing power output by minimizing current fluctuations and lowering the size of passive components. This results in improved transient performance responsiveness and achieving thermal distribution Hwu et al. [47]. The interleaved topology is characterized by its simplicity, which is a notable quality. In this topology, the cells of the IBC collectively carry the input current, resulting in tiny ripples in the current. This, in turn, helps to preserve the life span of fuel cell stacks Tomaszuk et al. [48]. IBC is comprised of “n” individual boost converters that are interconnected in tandem. There is an unlimited number of legs for the interleaved power branches in the simulation. As the number of phases rises, the system has a corresponding increase in complexity and maintenance challenges, as shown via real implementation. The drawback of using the interleaving strategy lies in the increase in complexity of the gate driving logic, as well as the significant influence on the dimensions and expenses of the gate drive Ramasamy et al. [49]. The shift in phase signal used to activate the gate switches is described:

(10) $$\theta =\frac{360{}^{\circ}}{n}$$

Let n be the selected no of phases for interleaved boost converter.

### 3.1 Two-phase interleaved boost converter

**Figure 4:**
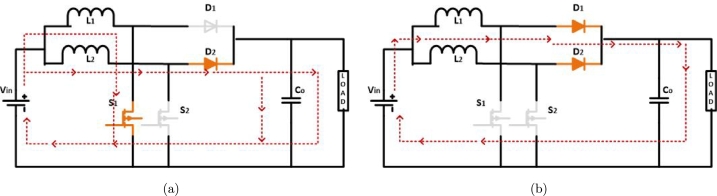
(a) *S*_1_ closed, (b) *S*_1_ and *S*_2_ are opened.

Operation of 2 *ϕ* IBC is splitting into 2 different states and comprises 2 different modes. Phase shift between each switch is precisely 180 degrees since the value of n is equal to two, as shown by Equation (10) as the operation is explained in Fig. 4 Nahar et al., Harinee et al., Alavi et al. [19], [20], [45].

**Mode 1:** When $S_{1}$ switch is closed and switch $S_{2}$ is open. The current flowing through $L_{1}$ started to increase, while $L_{2}$ started to release its charge to the load with $D_{2}$ running in forward bias, in Fig. 4(a).

**Mode 2:** While $S_{1}$ & $S_{2}$ are in the open position. Present electrical currents flowing through the output of the circuit connect $L_{1}$ and $L_{2}$ to the load, with $D_{1}$ and $D_{2}$ functioning as forward bias. This configuration is in Fig. 4(b).

#### 3.1.1 First modified-interleaved boost converter

**Figure 5:**
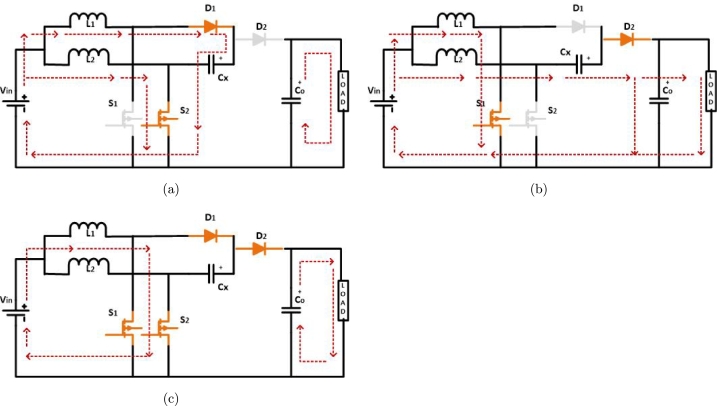
First modified-interleaved boost converter operation: (a) *S*_2_ closed, (b) *S*_1_ closed, and (c) *S*_1_ & *S*_2_ are closed.

An inherent flaw in the IBC design is the minimal voltage amplification. IBC structures may be paired with a voltage doubler to increase their voltage and gain. The first Modified-IBC circuit is the same as the regular IBC circuit, except it incorporates an additional capacitor, denoted as $C_{x}$ Chen et al. [21]. A duty cycle value is required to activate the IBC with a voltage doubler (0.5≤D≤1). According to Fig. 5, this architecture is partitioned into three modes.

**Mode 1:** While switch $S_{1}$ is in the OFF position, diode $D_{1}$ functions with forward bias, allowing the charging of $C_{x}$ from $L_{1}$, as shown in Fig. 5(a). The $C_{o}$ begins to discharge to provide power to load.

**Mode 2:** The $V_{i}n$, $L_{2}$, and $C_{x}$ series connection of components allows current to $C_{o}$ and load via $D_{2}$ while switch $S_{2}$ is in the OFF state and operating with a forward bias, illustrated in Fig. 5(b).

**Mode 3:** Switches are in the ON state, $L_{1}$ and $L_{2}$ are connected to $V_{i}n$ to begin the charging process Jang et al. [22] as in Fig. 5(c). The calculations of the first M-IBC parameters and equations are illustrated in Table 3, Inductors and capacitors are selected based on the values determined by these factors.

**Table 3:** Equations for designing components.

Types of Converters [ref]	Inductors		Capacitor	
	Equations	Parameters	Equations	Parameters
Two-phase IBC Nahar et al. Harinee et al. [19], [20], [45]	$L=\frac{V_{in}\cdot D}{f_{s}\cdot \Delta i_{L}}$	L1 = L2 = 100 μH	$C_{0}=\frac{V_{o}\cdot D}{f_{s}\cdot \Delta V_{o}\cdot R}$	C0 = 10 μF
First Modified IBC Jang et al. [22]	$L=\frac{V_{in}\cdot D}{f_{s}\cdot \Delta i_{L}}$	L1 = L2 = 100 μH	$C=\frac{V_{o}\cdot D}{f_{s}\cdot \Delta V_{o}}$$C_{0}=\frac{V_{o}\cdot D}{f_{s}\cdot \Delta V_{o}\cdot R}$	Cx = 40,000 μF Co = 10 μF
Second Modified IBC Zhou et al. [23]	$L=\frac{V_{in}\cdot D}{f_{s}\cdot \Delta i_{L}}$	L1 = L2 = 100 μH	$C_{1}=\frac{V_{o}\cdot D}{f_{s}\cdot \Delta V_{c}\cdot R\cdot {\left(1-D\right)}^{2}}$$C_{2}=\frac{V_{o}\cdot D}{f_{s}\cdot \Delta V_{o}\cdot R}$$C_{0}=\frac{V_{o}\cdot D}{f_{s}\cdot \Delta V_{o}\cdot R}$	C1 = 40,000 μF Co = 10 μF
Third Modified IBC Varesi et al. [24]	$L=\frac{V_{in}\cdot D}{f_{s}\cdot \Delta i_{L}}$ $L_{2}=\frac{V_{in}\cdot D\cdot (2-D)}{f_{s}\cdot \Delta i_{L}}$	L1 = L2 = 150 μH	$C_{1}=\frac{V_{o}\cdot D}{f_{s}\cdot \Delta V_{c}\cdot R\cdot {\left(1-D\right)}^{2}}$$C_{2}=\frac{V_{o}\cdot D}{f_{s}\cdot \Delta V_{o}\cdot R}$$C_{0}=\frac{V_{o}\cdot D}{f_{s}\cdot \Delta V_{o}\cdot R}$	C1 =C2 = 4 μF Co = 10 μF

#### 3.1.2 Second modified-interleaved boost converter

This research suggests incorporating a new voltage-doubler circuit into the existing IBC design. This circuit will increase the conversion voltage ratio and eliminate the need for a significant duty ratio in high step-up situations. Furthermore, the electrical pressure Compared to output voltage, power consumption of all power devices is much lower. This provides a way to better power devices with reduced voltage ratings. This converter is used using the Interleaved Boost Converter (IBC) topology, which consists of 2 power switches, $S_{1}$ & $S_{2}$. These switches function with a phase shift of 180 degrees and are divided into three distinct operating modes Zhou et al. [23].

**Mode 1:** As seen in 6(a) Figure, switches $S_{1}$ & $S_{2}$ are in closed position, causing inductors $L_{1}$ & $L_{2}$ to begin the process of charging. Furthermore, in this circuit, every power diode operates in reverse bias. The company begins to discharge and provide power to the load.

**Figure 6:**
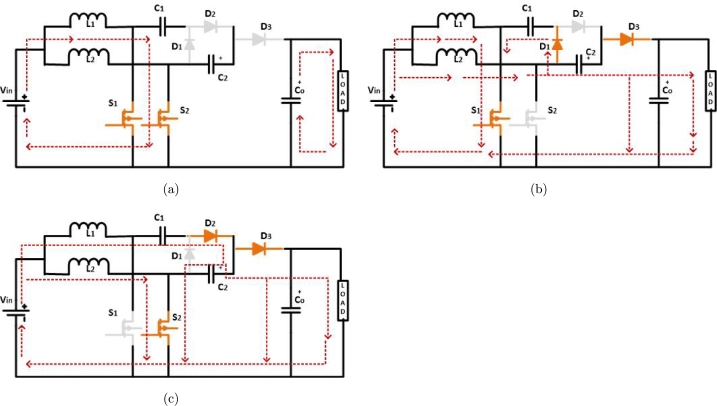
Second modified-interleaved boost converter operation: (a) *S*_1_ & *S*_2_ closed, (b) *S*_1_ closed, and (c) *S*_2_ closed.

**Mode 2:**$S_{1}$ switch is closed, inductor $L_{2}$ is drained to charge capacitor $C_{1}$. This is achieved by diode $D_{1}$ in forward Operation. Meanwhile, the inductor $L_{1}$ is still in the process of charging. This is seen in Fig. 6(b). Furthermore, the $L_{2}$ and $C_{2}$ components are connected in series to allow power to load and charge the capacitor $C_{o}$, and $D_{3}$ is in forward bias.

**Mode 3:**$S_{2}$ is closed, $L_{1}$ & $L_{2}$ undergo discharge to charge. $C_{2}$ through $D_{2}$ are in a state of forward bias, providing power to load and charging it. $C_{o}$ in $D_{3}$ is in forward bias, whereas $D_{2}$ is in the process of charging, as seen in Fig. 6(c).

#### 3.1.3 Third modified-interleaved boost converter

**Figure 7:**
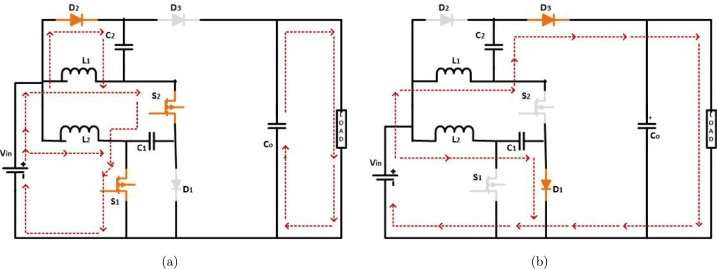
Third modified-IBC operation: (a) *S*_1_ & *S*_2_ closed, and (b) *S*_1_ & *S*_2_ opened.

A simple, easily controlled, and stress-reduced interleaved high-gain architecture is suggested in this research. Fig. 7 depicts the converter circuit, including 2 inductors ($L_{1}$ & $L_{2}$), three capacitors ($C_{1}$, $C_{2}$, & $C_{o}$) 2 switches ($S_{1}$ & $S_{2}$), and three diodes ($D_{1}$, $D_{2}$, and $D_{3}$). The switches operate in synchronization, enabling precise control of the converter, which is divided into two distinct operational modes Varesi, et al. [24].

**Mode 1:** The inductor $L_{1}$ begins to gather charges from the source $V_{i}n$ when switches $S_{1}$ and $S_{2}$ are closed, whereas the inductor $L_{2}$ begins to accumulate charges from $V_{i}n$ and $C_{1}$ when the switches are closed. While diode $D_{2}$ is in the forward bias state, capacitor $C_{2}$ is receiving a charge from both $V_{i}n$ and $C_{1}$. As shown in Fig. 7(a), the function $C_{o}$ is responsible for supplying the load with energy.

**Mode 2:** Switches $S_{1}$ & $S_{2}$ are opened. Inductor $L_{1}$ charges Capacitor $C_{1}$ via $D_{1}$ while operating in forward bias. Furthermore, inductor $L_{2}$, input voltage $V_{i}n$, and capacitor $C_{2}$ transfer energy to load, while the capacitor $C_{o}$, via the operation of a diode $D_{3}$, functions in a forward bias in Fig. 7(b).

The equations and values that are chosen for the components are shown in Table 3. The above properties of converters have been considered for the implementation of a new converter. The advantages of constructional, operationally desired converter have been introduced in the next section

## 4 Proposed converter

**Figure 8:**
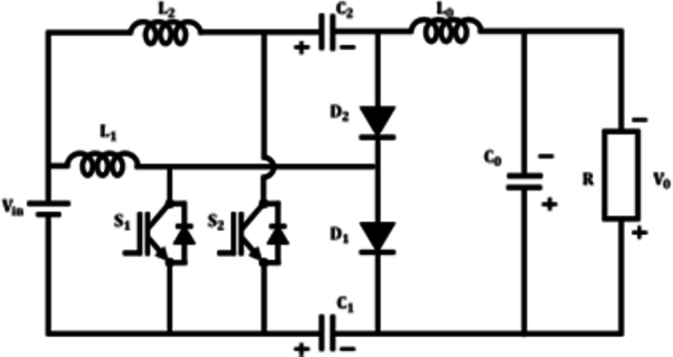
Interleaved boost-Cuk converter.

The fuel cell's voltage is inadequate for the efficient operation of the BLDC motor, therefore requiring the use of a power electronic converter. The proposed work involves the implementation of an interleaved Boost-Cuk converter, It integrates the distinctive characteristics of both boost and Cuk converters, to enhance voltage of a fuel cell. Fig. 8 illustrates the arrangement of planned IBC converter.

### 4.1 IBC converter modes of operation

**Figure 9:**
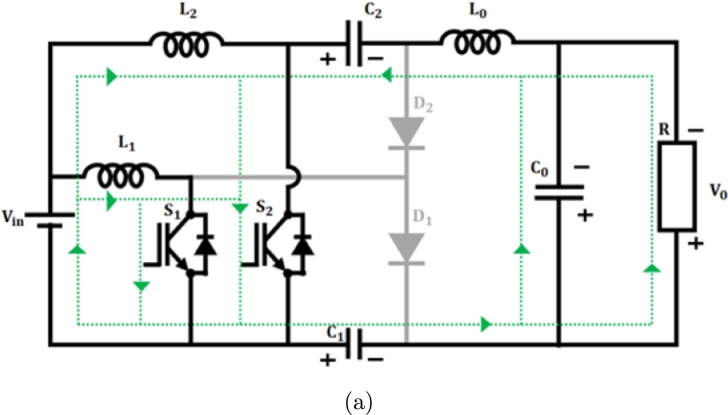

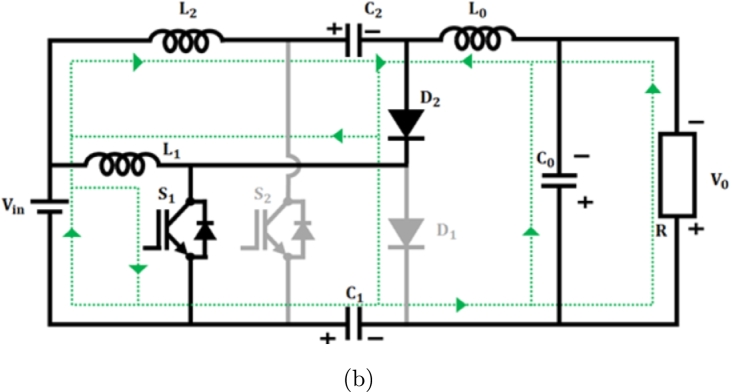

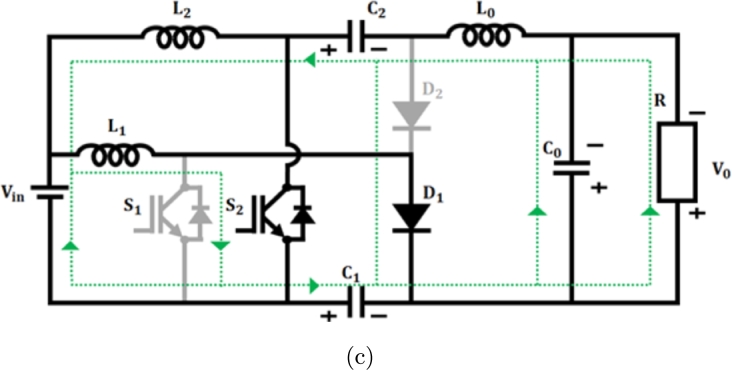
(a) *S*_1_ and *S*_2_ closed, (b) *S*_1_ closed (c) *S*_2_ closed.

**Mode 1:** Switches $S_{1}$ & $S_{2}$ are ON state, while diodes $D_{1}$ & $D_{2}$ turned OFF, as in Fig. 9(a). During this state, the inductor $L_{1}$ begins to charge by receiving voltage from the source $V_{i}n$. Similarly, capacitors connected in series $C_{1}$ and $C_{2}$ discharge and charge the output inductor $L_{0}$ by switch $S_{2}$. As a result, the output inductor current $i_{L_{0}}$ consistently rises.

(11) $$V_{in}=V_{L1}$$

(12) $$V_{in}=V_{L2}$$

(13) $$V_{L1}=V_{L2}$$

(14) $$V_{C2}+V_{L0}-V_{C0}-V_{C1}=0$$

(15) $$V_{C0}=V_{0}$$

**Mode 2:** In this manner, switch $S_{1}$ stays in the ON state, while switch $S_{2}$ becomes OFF, as shown in Fig. 9(b). In this scenario, the diode $D_{1}$ switches to the OFF state while the diode $D_{2}$ switches to the ON state. Consequently, the energy from the leaky inductor $L_{2}$ is detected by capacitor $C_{2}$ via use of a diode $D_{2}$, resulting in a decrease in current flowing through diode $i_{D_{2}}$. Consequently, output inductance $L_{0}$ charges the load constantly resulting in decreased inductor current $i_{L_{0}}$

(16) $$V_{L1}-V_{L2}-V_{C2}=0$$

(17) $$V_{L2}=V_{L1}-V_{C2}$$

(18) $$V_{L0}-V_{C0}-V_{C1}=0$$

**Mode 3:** During this scenario switch $S_{1}$ occupies OFF position and the switch $S_{2}$ goes ON as depicted in Fig. 9(c). Accordingly, $D_{1}$ diode goes ON and $D_{2}$ turns OFF. In this instance, energy from leakage inductance $L_{1}$ is absorbed by $C_{1}$ capacitor by $D_{1}$ diode, and the related current $i_{D_{1}}$ drops in linear. Therefore, the voltage across the switch $S_{1}$ becomes capped at $V_{C_{2}}$ As a consequence, the output inductor $L_{0}$ stores energy constantly resulting in increasing inductance current $i_{L_{0}}$.

(19) $$V_{in}-V_{L1}+V_{C1}=0$$

(20) $$V_{in}+V_{C1}=V_{L1}$$

(21) $$V_{C0}-V_{C2}-V_{L0}-V_{C1}=0$$

(22) $$V_{C1}-V_{L2}-V_{C2}-V_{L0}-V_{C0}=0$$

On subtracting Equation (15) from (16)

(23) $$V_{L1}-V_{L2}-2V_{C0}=0$$

Applying $V_{C0}=V_{0}$ and $V_{in}=V_{L2}$

(24) $$V_{L1}-V_{in}-2V_{0}=0$$

on applying inductor volt second balance equation

(25) $$(V_{\left(L_{1},S_{on}\right)})(DT)+(V_{\left(L_{1},S_{off}\right)})(1-D)T=0$$

by substituting Eq (5) & (20) in (21)

(26) $$V_{in}(DT)+(V_{in}+2V_{0})(1-D)T=0$$

(27) $$V_{in}DT+V_{in}T+2V_{0}T-V_{in}DT-2V_{0}DT=0$$

(28) $$V_{in}T+2V_{0}T-2V_{0}DT=0$$

(29) $$V_{in}+2V_{0}-2V_{0}D=0$$

(30) $$V_{in}-2V_{0}(1+D)=0$$

(31) $$V_{in}=2V_{0}(1+D)$$

Converter gain is expressed as

(32) $$M=\frac{V_{0}}{V_{in}}\;=\frac{1}{2(1+D)}$$

Improved proposed converter's voltage gain

**Figure 10:**
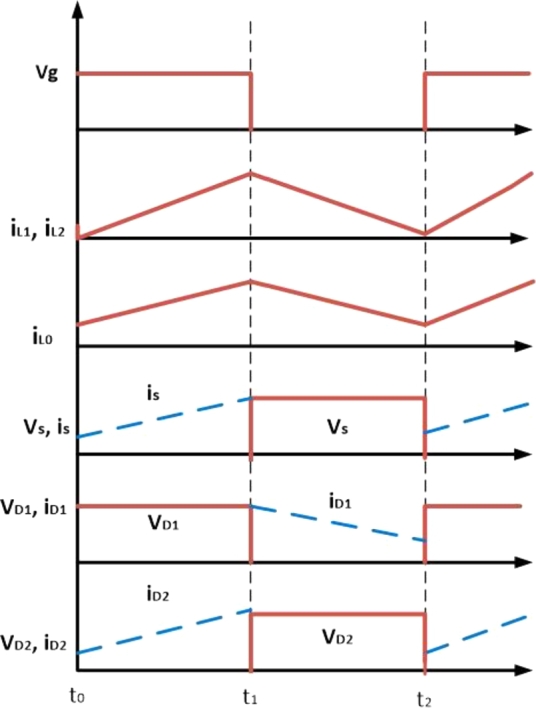
Waveform of proposed IBC converter's switching.

Fig. 10 shows switching waveform of proposed Converter

Generalized formula for component design

(33) $$Inductor=\frac{V_{in}D}{f\cdot k_{i}\cdot I_{in}}$$

(34) $$Capacitor=\frac{I_{out}(1-D)}{f\cdot k_{v}\cdot V_{out}}$$

**Voltage Stress:**

Voltage stress across capacitors $C_{1}$ & $C_{2}$ is expressed as;

(35) $$V_{vps}-C_{1}=\frac{1}{1-D}\;V_{in}=\frac{1}{3+D+2n}\;V_{0}=2.33\text{ V}$$

(36) $$V_{vps}-C_{2}=\frac{2}{1-D}\;V_{in}=\frac{1}{3+D+2n}\;V_{0}=3.20\text{ V}$$

Where n=1 and D=0.6

Voltage stress across Switches $S_{1}$ & $S_{2}$ is expressed as

(37) $$V_{vps-S_{1}}=V_{vps-S_{2}}=\frac{1}{1-D}V_{\text{in}}=\frac{1}{3+D+2n}\;V_{0}=55\text{ V}$$

Similarly, voltage stress across diode is given by,

(38) $$V_{vps-D_{1}}=\frac{1}{1-D}\;V_{in}=\frac{1}{3+D+2n}\;V_{0}=0.453\text{ V}$$

(39) $$V_{vps}-D_{2}=\frac{2}{1-D}V_{in}\;=\frac{2}{3+D+2n}\;V_{0}=0.49\text{ V}$$

**Loss Analysis:**

The losses across switches involve switching losses and conduction losses $C_{loss}$, and the expression is given by:

(40) $$P_{S}=I_{rms-S_{1}}^{2}r_{S_{1}}+\frac{f_{s}}{2}\left[V_{DS_{1}}I_{L_{1}}t_{off1}+V_{DS_{1}}^{2}C_{loss1}\right]+I_{rms-S_{2}}^{2}r_{S_{2}}+\frac{f_{s}}{2}\left[V_{DS_{2}}\left(I_{L_{1}}+I_{0}\right)t_{off2}+V_{DS_{2}}^{2}C_{loss2}\right]=1220\;\mathrm{mW}$$

Capacitor loss is expressed as:

(41) $$P_{C}=\sum_{k=1}^{2}I_{rms-C_{k}}^{2}r_{C_{k}}+I_{rms-C_{0}}^{2}r_{C_{0}}=69.5\text{ mW}$$

(42) $$E_{ON}=\frac{V_{IGBT}\cdot I\cdot t_{ON}}{6}$$

(43) $$E_{OFF}=\frac{V_{IGBT}\cdot I\cdot t_{OFF}}{6}$$

(44) $$P_{SW}=\frac{1}{T}(N_{ON}E_{ON}+N_{OFF}E_{OFF})$$

where V is Blocking voltage of switch, $t_{ON}$ is ON Time, $N_{ON}$ is no ⋅ of on time, $N_{OFF}$ is no ⋅ of off time from the eq the individual switch losses have been evaluated the losses of switches $S_{1}$ = 0.0029 W-s $S_{2}$ = 0.0030 W-s

## 5 Results

MATLAB environment constructed to simulate suggested topology. The design parameters considered for simulation are seen in Table 4.

**Table 4:** Specification parameters.

Specification	Parameters
Fuel cell Specifications	

Maximum Power	1 kW
Voltage	90 V
Current	11.11 A
Fuel Supply Pressure	1.5 Bar
Temperature	338 K

BLDC Motor specification	

Speed	3000 rpm
Load Inertia	9× 10^−4^ Nm^2^

**Figure 11:**
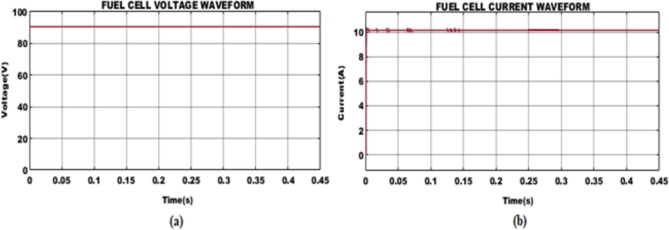
PEMFC (a) Voltage and (b) Current waveform.

**Figure 12:**
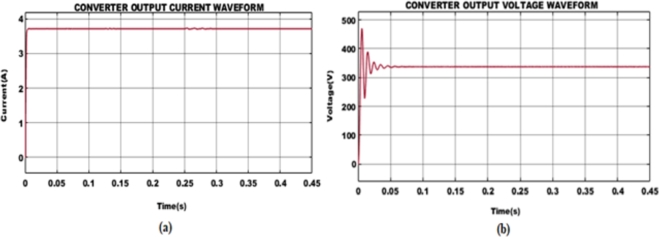
Interleaved Boost-Cuk converter (a) Current and (b) Voltage waveform.

Waveforms of current and voltage produced by PEMFC displayed in Fig. 11 and are impacted by features of load applied, operating circumstances, and the control method involved. A steady input fuel supply of 90 V is employed for efficient functioning of BLDC motor with a corresponding current of 11.11A which is obtained after 0.25 s with small variations at its first stage. The input fuel voltage demands essential boosting of voltage for optimal motor performance; therefore, the suggested work employs an interleaved Boost-Cuk converter. The waveforms displaying converter current, and voltage are presented in Fig. 12. A stable current of 3.7A is maintained constant without any aberrations. Similarly, on the voltage side, with variations in the beginning, a steady DC supply of 350 sV is established after 0.1 s and maintained further.

The key waveforms observed from BLDC motor such as motor current, back EMF, motor speed, and torque for varying speeds are depicted in Fig. 12.

**Figure 13:**
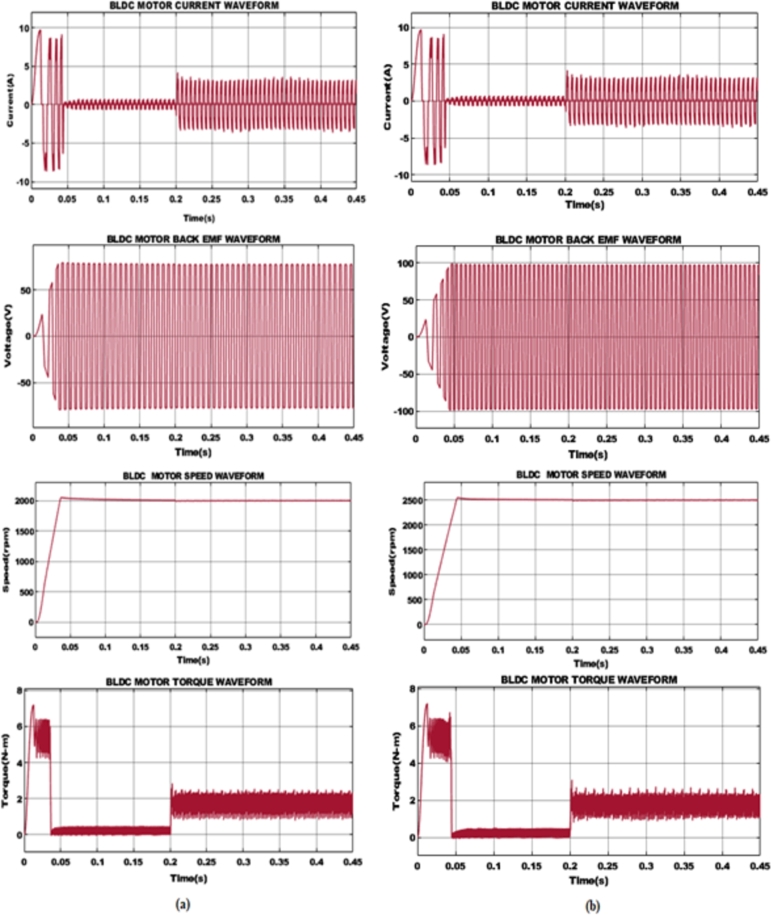
For speed (a) 2000 rpm and (b) 2500 rpm with load 0.2 s 1.5 Nm.

**Case 1: BLDC motor with 2000rpm speed:** The electrical current flowing through the windings of BLDC motor is depicted Fig. 13 (a), where the current varies in general and becomes stabilized. When load is supplied at 0.2 s the functioning of the motor normally shows a sinusoidal waveform which relies on the setup of the BLDC motor. A 75 V back EMF is attained after 0.5 s and thereafter maintained constant. Following, when power is provided to BLDC motor, speed rises gradually and achieves a steady state value of 2000rpm after 0.1 s. The torque of the BLDC motor reflects the twisting force created and displays changes of 1.5 Nm when the load is introduced at 0.2 s and held further constant due to no other variations in load circumstances.

**Case 2: BLDC motor with 2500rpm speed:** Similar to case 1, case 2 is based on a BLDC motor with a speed of 2500rpm as seen in Fig. 13(b). The current flowing on the BLDC motor displays distortions in its early stage and eventually becomes normalized. After the load is applied at 0.2 s a stable current is maintained. Likewise, the back EMF voltage of 98 V is created by the BLDC motor and kept steady throughout the system. On the other hand, when power is injected the speed of the BLDC motor displays changes by gradually growing in speed and maintained at 2500rpm and tends to continue further. Finally, a torque value of 1.5 Nm is obtained while the motor functions at a speed of 2500rpm with early aberrations in torque value at the beginning.

The comparison of converters in terms of component count and efficiency depicted in Table 5. It is observed that proposed converter achieves improved efficiency of 94%, while the 2 *ϕ* IBC Nahar et al. [19] 1st-M IBC Jang et al. [22] Zhou et al. [23] Varesi et al. [24]. interleaved Boost Li et al. [42] and interleaved Cuk Joseph et al. [43] converter shows reduced efficiency. To validate construction and operation logistics of proposed converter, hardware has been implemented for 1300 w.

**Table 5:** Comparison of converter.

Converters	No. of Switches	No. of Capacitors	No. of Diodes	No. of Inductors	Voltage gain	Efficiency (%)
2 *ϕ* IBC [19]	2	1	2	2	1⋅ 11	97⋅08
1st-M IBC [22]	2	2	2	2	1⋅ 11	98⋅36
2nd-M IBC [23]	2	3	3	2	1⋅ 11	98⋅99
3rd-M IBC [24]	3	3	3	2	1⋅ 11	99⋅09
Interleaved Boost [50]	2	2	3	2	5⋅83	91.7
Interleaved Cuk [51]	2	4	2	4	5	93
Proposed	2	2	2	3	3⋅8	94

## 6 Hardware setup

**Figure 14:**
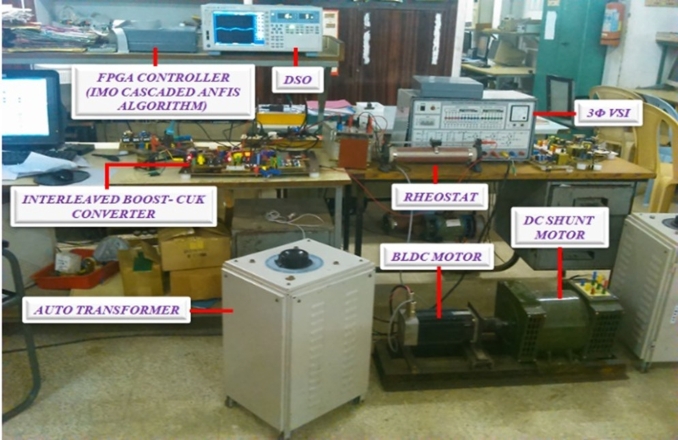
Hardware setup.

Fig. 14, shows the hardware setup.

**Figure 15:**
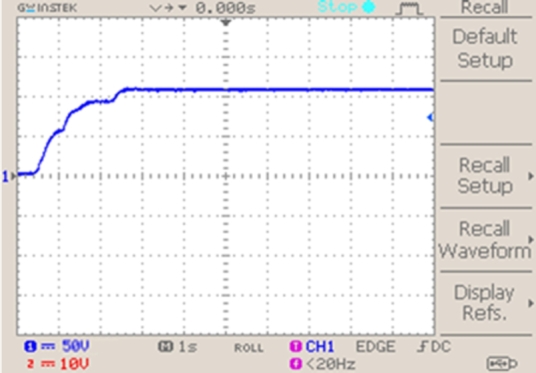
Voltage of fuel cell.

Fig. 15 demonstrates voltage across fuel cell and shows that a value of 110 V is maintained.

**Figure 16:**
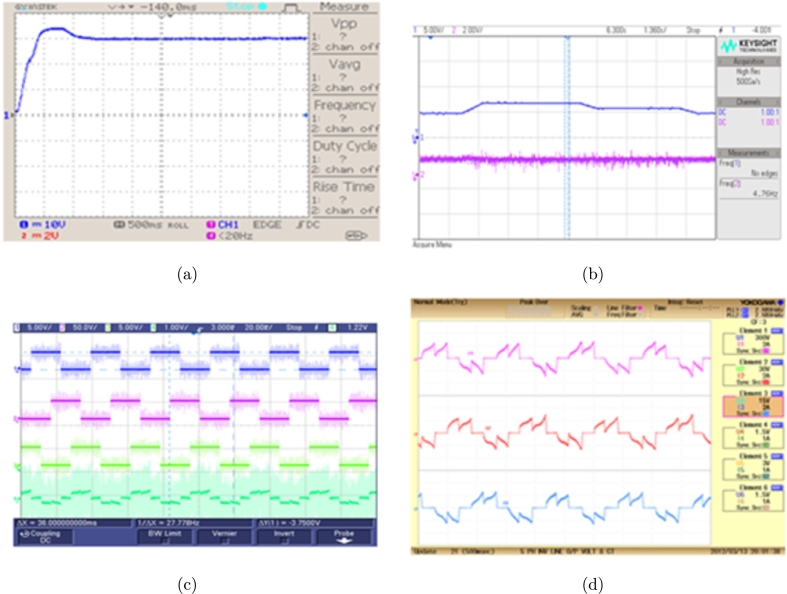
BLDC motor (a) Interleaved boost Cuk converter output voltage (b) Torque waveform (c) Hall sensor signal and stator current waveform and (d) Output current waveform.

Fig. 16(b) shows the BLDC motor's torque waveform, whereas Fig. 16(c) shows the waveforms for signals from the hall sensor and stator current. The position of rotor affects the rotor position measured by hall sensors and the ON/OFF state of electronic commutator switches. The current for each of the three phases of the BLDC motor is controlled using Interleaved Boost-Cuk, is in Fig. 16(d). The converter output characteristics are verified to claim the converter operation Fig. 16 from (a)-(d) it can be claimed that Proposed converter is capable of providing the required operational features to drive the BLDC with the speed of 2000, 2500 rpm.

**Figure 17:**
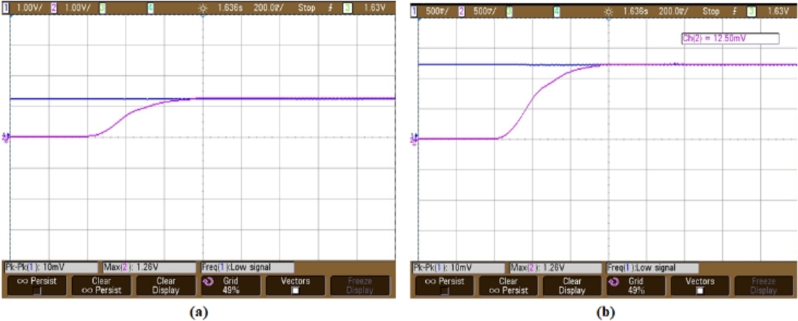
For speed of waveform using PI controller (a) 1500 rpm and (b) 2500 rpm.

Fig. 17(a) illustrates speed waveform of BLDC motor controlled by a PI controller, showcasing speeds of 1500 rpm and 2500 rpm. The waveforms demonstrate a rapid initial acceleration, subsequently transitioning to a steady, constant speed. Similar to the scenario with reduced speed, the waveform depicted in Fig. 17(b) demonstrates the motor's performance as it reaches the target speed of 2500 rpm. This indicates the speed performance of a motor equipped with a PI controller, ensuring reliable and efficient control across various operating conditions.

## 7 Conclusion

The research has focused on the interleaved converter's design and operational attributes. Initially, the research has provided the constructional details of interleaved converters with the review of the recent four boost-supported interleaved converter logistics and a comparison of the converter attributes has been presented and necessary features of the converter have been explained based on that in this article the suitable constructional featured converter has been introduced for the solution of higher parts count and efficiency with three modes of operations. To validate the constructional features simulation has been performed to understand the component and output characteristics of 350 V for BLDC motor application. To validate operational characteristics of proposed converter the simulation results have been presented and the specifications. The operational analysis of the BLDC motor has been presented in terms of speed regulation and torque. From the exhaustive analysis of proposed converter, it can concluded proposed converter is capable of driving BLDC motor with an input voltage of 35ov, and the BLDC drive is controlled with different speed regulations with a better torque ripple of 1.5 Nm with the efficiency of 94% then according to the operational characteristics it can be claimed that proposed converter is a better solution for commercial BLDC drive application, respectively.

## CRediT authorship contribution statement

**P.M. Preethiraj:** Writing – review & editing, Writing – original draft, Methodology, Investigation, Formal analysis, Conceptualization. **J. Belwin Edward:** Writing – review & editing, Writing – original draft, Visualization, Validation, Supervision, Resources, Methodology, Investigation, Conceptualization.

## Declaration of Competing Interest

The authors declare that they have no known competing financial interests or personal relationships that could have appeared to influence the work reported in this paper.
